# Effects of recent cannabis consumption on eye-tracking and pupillometry

**DOI:** 10.3389/fnins.2024.1358491

**Published:** 2024-04-09

**Authors:** Mohammad N. Haider, Daniel Regan, Mahamudul Hoque, Fahed Ali, Andrew Ilowitz

**Affiliations:** ^1^Department of Orthopedics, Jacobs School of Medicine and Biomedical Sciences, State University of New York at Buffalo, Buffalo, NY, United States; ^2^University Concussion Management Clinic and Research Center, UBMD Orthopedics and Sports Medicine, Buffalo, NY, United States; ^3^Department of Biological Sciences, Jacobs School of Medicine and Biomedical Sciences, State University of New York at Buffalo, Buffalo, NY, United States; ^4^Integral Health, Westport, CT, United States

**Keywords:** cannabis, eye tracking, pupillometry, oculogica, EyeBOX

## Abstract

**Introduction:**

Cannabis consumption is known to immediately affect ocular and oculomotor function, however, cannabis consumption is also known to affect it for a prolonged period of time. The purpose of this study is to identify an eye tracking or pupillometry metric which is affected after recent cannabis consumption but is not confounded by cannabis consumption history or demographic variables.

**Methods:**

Quasi-experimental design. Participants who would consume inhalable cannabis (*n* = 159, mean age 31.0 years, 54% male) performed baseline neurobehavioral testing and eye-function assessments when they were sober. Eye function assessments included eye-tracking [gaze (point of visual focus), saccades (smooth movement)] and pupillometry. Participants then inhaled cannabis until they self-reported to be high and performed the same assessment again. Controls who were cannabis naïve or infrequent users (*n* = 30, mean age 32.6 years, 57% male) performed the same assessments without consuming cannabis in between.

**Results:**

Cannabis significantly affected several metrics of pupil dynamics and gaze. Pupil size variability was the most discriminant variable after cannabis consumption. This variable did not change in controls on repeat assessment (i.e., no learning effect), did not correlate with age, gender, race/ethnicity, or self-reported level of euphoria, but did correlate with THC concentration of cannabis inhaled.

**Discussion:**

A novel eye-tracking metric was identified that is affected by recent cannabis consumption and is not different from non-users at baseline. A future study that assesses pupil size variability at multiple intervals over several hours and quantifies cannabis metabolites in biofluids should be performed to identify when this variable normalizes after consumption and if it correlates with blood THC levels.

## Introduction

Cannabis, and its primary derivative Delta 9 – tetrahydrocannabinol (Δ9-THC), is a psychoactive compound that produces feelings of euphoria, dysphoria, sedation, difficulty concentrating, impaired short-term memory and impaired body movement (balance and fine psychomotor control).(Ashton, 1999). Cannabis use, especially those with higher THC content, is also associated with neuropsychological disorders (Martinotti et al., 2011, 2012). Historically, cannabis and its derivatives have been classified as a Schedule I controlled substance in the United States (Mead, 2019), but recent changes in laws have approved its medical and recreational use in several States (Smart and Pacula, 2019; Blanchette et al., 2022). With its increased popularity of legal and illegal consumption (Lu et al., 2023), there is an increased interest in identifying methods to identify if a person is currently under the influence of cannabis or not, such as at work (Macdonald et al., 2010) or while driving (Martin et al., 2017). According to National Institutes of Justice, biofluid quantification of cannabis metabolites are unreliable markers of recent use (National Institute of Justice, 2021), which may be attributed to their complex metabolism and lipophilic nature (Chayasirisobhon, 2020), different modes of consumption (Newmeyer et al., 2017; Zipursky et al., 2020), as well as the use of synthetic forms of cannabis (Tagen and Klumpers, 2022).

One possible indirect measure, i.e., those which are not quantifying cannabis or its metabolites in biofluids, may be related to the eyes. Drug Recognition Experts already use some aspects of ocular function, such as horizontal gaze nystagmus during a field sobriety test, as indirect markers of cannabis intoxication as part of their comprehensive assessment (Hartman et al., 2016). Rapid and portable, technology-assisted measurements of different aspects of eye function have been used to diagnose abnormalities in a multitude of neurological conditions, such as traumatic brain injuries (Samadani et al., 2016; Oldham et al., 2021), degenerative brain disorders (Lin et al., 2015) and cognitive impairment (Tao et al., 2020), so it may be possible to use it for recent cannabis consumption as well. However, published literature on how it affects the eyes is conflicting. Some studies have found that acute cannabis consumption produces a dose-related constriction of the pupil (Hepler et al., 1972; Brown et al., 1977) whereas others report the opposite (Stark et al., 2003). Another important confounder is that cannabis use can also affect the eyes for a prolonged period of time. Studies have found that deficits in saccadic control and visuospatial memory (Huestegge et al., 2009, 2010) were observable more than 24 h after heavy cannabis users abstained from drugs and alcohol. Another study found that a higher lifetime consumption of cannabis had a negative association with blinking rates in heavy-users who were not under the influence at the time (Kowal et al., 2011), which the authors theorized may be due to impairment of dopaminergic pathway transmission.

To improve our understanding on which technology-assisted eye measurements are affected by recent consumption only, we performed an observational, quasi-experimental study where we measured multiple aspects of eye function before and after participants inhaled their own recreational cannabis. We also assessed the same parameters in a control sample who were cannabis naïve or infrequent consumers at two timepoints. Our specific interests were to identify which eye tracking metrics were significantly altered in cannabis users immediately after consuming inhaled cannabis that were not different from controls when users had abstained for several hours. Results from this pilot study will be used to design future trials.

## Methods

The protocol for this study was reviewed and approved by WIRB (IRB number: 20204456). Participants were recruited from medical cannabis dispensaries and word-of-mouth in Buffalo, NY and Westport, CT from February 2021 to October 2021. Interested participants called on the provided number and a member of the research team explained the study and screened for eligibility. If eligible, participants came to the research office and were provided with a consent form that further explained the purpose of the study. Participants were allowed to ask more questions and signed consent was obtained in a HIPAA compliant setting from all participants.

### Participants

The inclusion criteria for the Cannabis Group were the following: (1) aged 21–55 years; (2) were sober at the time of baseline assessments and had not consumed inhaled cannabis within the past 6 h or edible cannabis for the past 24 h; and (3) consented to participate in the study with their own inhaled cannabis. Participants were excluded if they self-reported to be under the influence of alcohol or narcotics during the research assessment, blind in one or both eyes, were unable to open their eyes, had a history of unresolved strabismus or oculomotor dysfunction, and/or did not have a safe mode of transport after the study. The inclusion criteria for the Control Group were the following: (1) aged 21–55; and (2) were cannabis naïve or were infrequent (< 1/month) consumers of cannabis. The exclusion criteria were identical to the Cannabis Group except for the safe mode of transportation.

### Cannabis consumption

Cannabis consumption was limited to smoking flower or inhaling vapor from vape pens due to the longer duration of onset of edible cannabis (Zipursky et al., 2020). Participants were asked to bring their own Δ9-THC products for consumption. The rationale for asking participants to consume their own cannabis was because it makes the results of our study more generalizable to the public (Mead, 2019; ElSohly et al., 2021). All participants in the Cannabis Group were given the same basic instructions: to consume cannabis *until they felt high*, which is a common term used for cannabis-induced euphoria. Percent THC was recorded from cannabis containers. Consumption was self-regulated, and participants were asked to consume whatever they were comfortable with. Participants had the option to consume cannabis with tobacco if preferred. The Cannabis Group was also asked to grade their level of euphoria during the post-cannabis assessments into the following categories: (a) slightly high; (b) moderately high; or (c) very high.

### Data collection procedure

All experimental procedures were performed in a private office setting which included a desk for paperwork, neurocognitive testing and eye-tracking assessment, and an open 9×9 foot area for balance testing. Assessments were done in a distraction-free setting with uniform lighting. After providing consent, all participants filled out a demographics and cannabis consumption history questionnaire and performed the following assessments in order: (1) vital sign assessment; (2) Digit Symbol Substitution Test (DSST); (3) balance assessment; (4) Paced Visual Serial Addition Task (PVSAT); (5) eye-tracking. Cannabis Group participants then consumed cannabis either within the office or in an outdoor private courtyard based on their preference. The examiner waited until participants reported they were high and were ready to perform the repeat assessment in the exact same order. Time duration from the onset of inhaling cannabis to starting the repeat assessment was recorded. Percent THC and mass of product used was recorded for inhaled flower but could not be recorded for vaporized pens, hence they were not used for analysis. The Control Group repeated the same assessments in the same order after 15 min without consuming cannabis in between. Participants were provided with a $75 Amazon gift card after the assessments were complete.

### Main outcome measures

No biofluid assessment of cannabis was performed in this study which is described in detail at the end of the manuscript. Before recruitment started, a standardized protocol for all assessment procedures was developed and agreed upon by the lead investigators of both sites (MNH from NY and AI from CT).

#### Vital signs

Blood pressure and pulse was obtained in a seated position using an automated wrist sphygmomanometer (Omron Healthcare Co., Japan) and temperature was obtained using a non-contact infrared body thermometer (Hetaida Technology Co., China).

#### Digit symbol substitution test

The DSST is a paper and pencil neurocognitive test that assesses motor speed, attention and visuospatial memory, and the ability to write and draw (i.e., basic manual dexterity) (Aster et al., 2006). To avoid the learning effect, participants performed List A at baseline, and then List B at the repeat assessment. To avoid the ceiling effect, the time for each test was reduced to 90 s (standard: 120 s).

#### Balance test

The Walk and Turn (WAT) and One Leg Stand (OLS) tests were used to assess coordination and balance (Downey et al., 2016). They are two of several field sobriety tests developed by the National Highway Traffic Safety Administration and are also called the Divided Attention Task A and B, respectively. For the purposes of roadside testing, the WAT and OLS tests are considered indicators of impairment if there are two or more abnormalities in either of these tests. Prior to starting recruitment, all researchers who graded the balance test reviewed standardized instructions and practiced the test together to ensure that grading was standardized. The horizontal gaze nystagmus component of Divided Attention Task was excluded due to lack of agreement between a trained clinician with expertise in oculomotor exams (MNH) and research assistants (DR and MH). Abnormal clues on WAT included incorrect number of steps in either direction, starting too early, stopping while walking, stepping off the line, raising arms for balance, unable to touch heel-to-toe, and having trouble turning (max = 8). Abnormal clues on OLS included putting one foot down, hopping, raising arms more than 6 inches from body or losing balance (max = 4).

#### Paced visual serial addition task

While remaining seated and following instructions and a 30-s (10 digit) practice, participants completed a 3-min computerized PVSAT, which assesses attention and information processing speed (Fos et al., 2000). Participants are presented numbers on a screen in 3 s intervals and are asked to sum the current and previous numbers.

#### Eye-tracking

The Oculogica EyeBOX (Oculogica Inc., New York, NY) was used in this study (Samadani et al., 2016; Zahid et al., 2018). Participants sat on a chair with their heads about 40 cm from a 12.3-inch screen. A list of all 71 calculated metrics is provided in Supplementary File 1. Participants watched a 90 s video clip of a movie on a 2-inch by 2-inch window which moved in a square frame along the border of the screen for 2 cycles. Participants were asked to keep their heads stationary and track the video with their eyes. While watching the video, the EyeBOX camera was used to record where the right and left pupils were focused on with a sampling rate of 167 Hz (Supplementary Figure S1). The EyeBOX is a reliable infra-red camera that has been used in clinical eye-related research and a detailed explanation of how the data is obtained, processed and visualized has been published (Samadani et al., 2016). The EyeBOX performs a quality screen immediately after the tests is done. If the quality of scan was below acceptable, then participants were asked to repeat the scan.

### Statistical analysis

No *a priori* sample size estimation was performed due to the pilot nature of the study and Cannabis Group participants were recruited at a 4:1 ratio due to limitations in funding. The normality of continuous variables was assessed and most of them were found to not be normally distributed (Shapiro–Wilk *p* < 0.05). Demographics, cannabis consumption history, vital signs and neurobehavioral tests were compared at the pre-and post-consumption timepoints with controls. Mann–Whitney U test was used to compare continuous variables between groups and a χ^2^ test was used for categorical variables. Cannabis Group’s eye-tracking metrics before and after consumption and Control Group at first and repeat visit were compared using a paired t-test. Effect sizes were calculated. Additional analysis was performed for the most discriminant variable and correlations (Spearmans) with demographics and cannabis-related variables were performed. A *p*-value of <0.05 was considered statistically significant. A Bonferroni correction was applied for multiple comparisons only and a *p*-value of <0.017 was considered significant for these tests (3 pairs, 0.05 ÷ 3). All analyses were performed using SPSS Version 29 (IBM Corp, Armonk, NY) (IBM Corp, 2021).

## Results

A total of 193 participants were interested in performing the study. Two participants did not want to perform the study after inquiring about it, 1 participant was not eligible due to an unresolved oculomotor dysfunction (amblyopia), and 1 participant provided consent but had to leave before starting any assessments and did not reschedule their visit. Hence, a total of 189 participants performed the study are included in this analysis, with 159 participants in the Cannabis Group and 30 participants in the Control Group. No differences were seen in demographics except that the Cannabis Group consumed cannabis more frequently than the Control Group. No adverse events were reported during the entire study, however, one relatively young participant (23 y/o male) had slightly elevated blood pressure at baseline (161/89 mmHg) which increased to 215/152 mmHg after smoking. The participant did not report any hypertensive symptoms (headaches, dizziness), had never been diagnosed with hypertension, and were not aware if their blood pressure increases this much every time they consumed cannabis. This participant was recommended to follow-up with their primary care physician to address any underlying causes for hypertension and continued with the research assessments (see Table 1).

**Table 1:** Groupwise participant demographics and cannabis use history.

Demographics	Cannabis Group	Control Group	*p*-value
Sample size	159	30	–
Age, mean (95% CI)	30.97 (29.43, 32.50)	32.56 (28.50, 36.61)	0.446
Gender, *n* (%)			0.609
Male	85 (53.5%)	17 (56.7%)	
Female	71 (44.7%)	13 (43.3%)	
Other/Non-binary	3 (1.9%)	0 (0%)	
Race, n (%)			0.496
White	124 (78.0%)	27 (90.0%)	
Black	18 (11.3%)	1 (3.3%)	
Asian	10 (6.3%)	2 (6.7%)	
Native American	2 (1.3%)	0 (0%)	
Other or Not Reported	5 (3.1%)	0 (0%)	
Hispanic or Latino, *n* (%)	16 (10.1%)	3 (10.0%)	> 0.999*
Education, *n* (%)			0.414
Did not complete High school	1 (0.6%)	1 (3.3%)	
High school only	14 (8.8%)	2 (6.7%)	
College student	40 (25.2%)	5 (16.7%)	
College Graduate	77 (48.4%)	14 (46.7%)	
Post-graduate	27 (17.0%)	8 (26.7%)	
Cannabis use frequency, *n* (%)			**< 0.001**
Cannabis naïve	0 (0%)	16 (53.3%)	
< 1/month	4 (2.5%)	14 (46.7%)	
1/month to less than 1/week	10 (6.3%)	0 (0%)	
1–6 times/week	44 (27.7%)	0 (0%)	
Every day	101 (63.5%)	0 (0%)	

Bolded values indicate a significant finding; CI: confidence interval; *p-value from Fishers Exact test.

One hundred and thirty-two participants (83.0%) in the Cannabis Group smoked flower and 27 (17.0%) inhaled vapor pens. Eighteen participants who smoked cannabis flower consumed it with tobacco (i.e., spliff or blunt). Twenty-five (15.7%) participants reported to be slightly high, 92 (57.9%) reported to be moderately high, and 42 (26.4%) reported to be very high. Mean THC concentration of product used was 30.0% ± 15.3 (median = 23%, range = 12–89%). Groupwise physiological and neurobehavioral assessments before and after consumption are presented in Table 2. When comparing Cannabis Pre-consumption to Control Baseline, no differences were seen in any assessments. After consumption, the Cannabis Group had higher pulses and blood pressures, and performed worse on the PVSAT, WAT and OLS than Control’s repeat assessment. The median time duration between assessment was 15 min for the Control Group but ranged from 15 min to 1 h and 30 min for the Cannabis Group.

**Table 2:** Groupwise physiological and neurobehavioral assessments at baseline and after consumption.

	Cannabis pre-consumption	Control baseline	*p*-value
Temperature in F	98.29 (98.20, 98.39)	98.26 (98.14, 98.38)	0.557
Pulse in bpm	74.58 (72.51, 76.65)	71.73 (68.10, 75.37)	0.256
Systolic BP in mmHg	121.93 (119.31, 124.55)	118.27 (113.38, 123.15)	0.287
Diastolic BP in mmHg	77.30 (75.68, 78.92)	74.20 (70.99, 77.41)	0.082
DSST Total Correct	63.30 (61.16, 65.44)	59.47 (54.73, 64.20)	0.117
PVSAT Correct	45.75 (43.88, 47.61)	48.27 (43.93, 52.60)	0.199
PVSAT Incorrect	6.22 (5.37, 7.08)	5.23 (3.09, 7.38)	0.218
PVSAT Missing	8.03 (6.83, 9.23)	6.50 (3.79, 9.21)	0.211
PVSAT Reaction Time in seconds	1.65 (1.61, 1.69)	1.60 (1.51, 1.69)	0.241
WAT, *n* (%)			0.168
Two or more clues	13 (8.3%)	1 (3.3%)	
One clue	46 (29.5%)	5 (16.7%)	
No mistakes	97 (62.2%)	24 (80.0%)	
OLS, *n* (%)			0.459
Two or more clues	7 (4.5%)	0 (0%)	
One clue	13 (8.4%)	2 (6.7%)	
No mistakes	135 (87.1%)	28 (93.3%)	

	Cannabis post-consumption	Control repeat	*p*-value^a^	*p*-value^b^
Temperature in F	98.39 (98.27, 98.50)	98.24 (98.12, 98.35)	0.373	0.224
Pulse in bpm	93.47 (90.58, 96.37)	70.77 (66.73, 74.80)	**< 0.001**	**< 0.001**
Systolic BP in mmHg	126.23 (123.46, 129.01)	117.20 (113.08, 121.32)	**0.003**	**0.027**
Diastolic BP in mmHg	80.58 (78.68, 82.49)	75.83 (72.42, 79.25)	**0.030**	**0.010**
DSST Total Correct	65.43 (63.10, 67.77)	62.77 (57.33, 68.20)	0.228	0.184
PVSAT Correct	48.08 (46.36, 49.79)	52.29 (48.57, 56.00)	**0.017**	0.070
PVSAT Incorrect	5.60 (4.79, 6.40)	3.68 (2.16, 5.20)	**0.043**	0.294
PVSAT Missing	6.33 (5.26, 7.40)	4.04 (1.45, 6.63)	**0.009**	**0.037**
PVSAT Reaction Time in seconds	1.58 (1.55, 1.62)	1.53 (1.45, 1.61)	0.245	**0.015**
WAT, *n* (%)			**< 0.001**^**c**^	**< 0.001**^**c**^
Two or more clues	37 (23.3%)	2 (6.7%)		
One clue	57 (35.8%)	4 (13.3%)		
No mistakes	65 (40.9%)	24 (80.0%)		
OLS, *n* (%)			**0.028**^**c**^	**< 0.001**^**c**^
Two or more clues	26 (16.6%)	1 (3.3%)		
One clue	28 (17.8%)	2 (6.7%)		
No mistakes	103 (65.6%)	27 (90.0%)		

Bolded values indicate a significant finding; values are presented as means (95% confidence intervals) or *n* (%). BP, blood pressure; DSST, digit symptom substitution test; PVSAT, paced visual serial addition task; WAT, walk and turn; OLS, one leg stance.

a *p*-value from Mann Whitney U test comparing Cannabis Post-consumption to Control Repeat.

b *p*-value from paired *t*-test comparing Cannabis Pre-to Post-consumption.

c *p*-value from chi-squared test.

Supplementary Table S1 presents the eye-tracking and pupillometry comparison between timepoints in the Control Group. Out of 71 eye tracking metrics, only 1 was different between timepoints after correcting for multiple comparisons: Vertical Saccade Travel Mean for the left eye (*p* = 0.005). The effect size for this change was 0.717, which corresponds to a medium (*d* < 0.8) effect (Brydges, 2019). This variable was not different when compared to the Cannabis Group when they were (*d* = −0.110, *p* = 0.132) or were not (*d* = −0.075, *p* = 0.442) under the influence. Due to minimal differences in the Control Group between timepoints, only the comparison between Cannabis Group and Control Baseline are described in subsequent comparisons.

**Figure 1:**
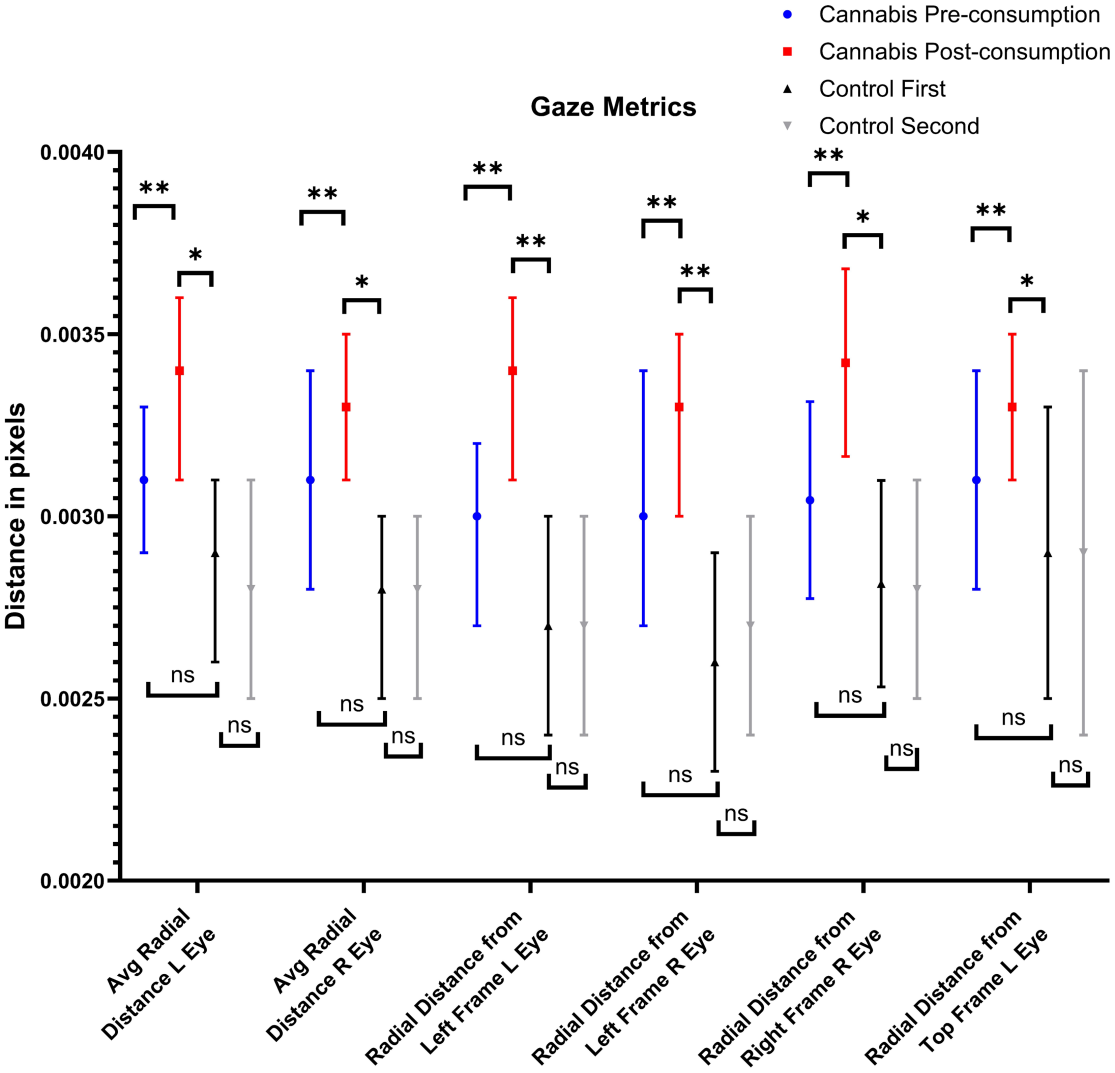
Gaze metrics that were affected by recent cannabis consumption. NS, not significant; ^*^: *p* < 0.05; ^**^: *p* < 0.017 (i.e., after correction for multiple comparisons).

Supplementary Tables S2a,b present all groupwise eye-tracking metrics and results from comparisons, respectively. When comparing gaze between Cannabis and Control groups, Cannabis Pre-consumption had higher mean path departure in their left eye (number of times they looked away, *p* = 0.050) and greater variance in the Y-axis when following the bottom part of the frame (*p* = 0.038) compared to Controls, but these were not statistically significant after correcting for multiple comparisons. Several differences were observed when comparing Cannabis Post-consumption to Cannabis Pre-consumption and Controls. Figure 1 presents the gaze metrics that were significantly different between Cannabis Pre-consumption to Cannabis Post-consumption and Cannabis Post-consumption to Controls, but not between Cannabis Pre-consumption to Controls. This includes 6 of the total 51 (11.8%) gaze-related metrics. All variables that were significantly different were related to abnormal distances between expected and actual point of visual focus. Specifically, the distance between the actual gaze position and the expected gaze position increased after cannabis consumption in the left, right and top side of the frame. Of note, although the comparison between Cannabis Pre-consumption and Controls First assessment was not statistically different, cannabis user’s mean values were higher than controls before consumption.

**Figure 2:**
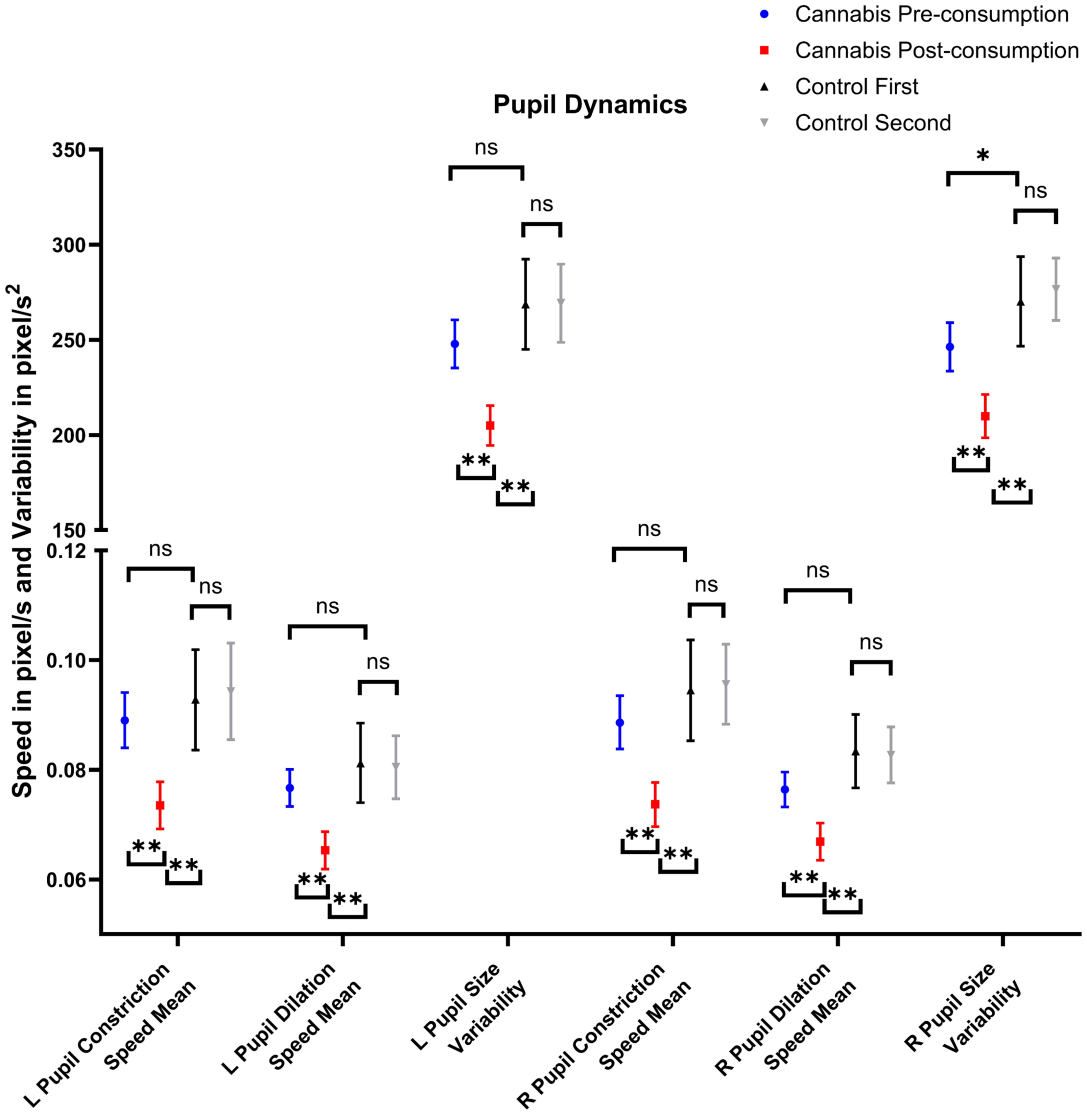
Pupil dynamics that are affected by recent cannabis consumption. NS, not significant; ^*^: *p* < 0.05; ^**^: *p* < 0.017 (i.e., after correction for multiple comparisons).

Supplementary Tables S3a,b presents groupwise pupil dynamics and results from comparisons, respectively. Unlike gaze metrics, several differences were observed in pupil metrics between Cannabis Pre-consumption and Controls. Cannabis Pre-consumption had larger mean (*p* = 0.018, 0.040), maximum (*p* = 0.018, 0.063) and minimum (*p* = 0.018, 0.044) left and right pupil sizes than Controls, but all of these were not significant after correcting for multiple comparisons. Figure 2 presents the pupillometry metrics that were significantly different between Cannabis Pre-consumption to Cannabis Post-consumption and Cannabis Post-consumption to Controls, but not between Cannabis Pre-consumption to Controls. This includes 6 out of the total 20 (30%) pupil-related metrics. These variables were related to pupil dynamics instead of pupil size. Cannabis consumption caused a reduction of right and left pupil dilation and constriction speeds. The variable with the largest effect was right and left pupil size variability, which is the root mean square of successive differences in pixels/s^2^ and is explained in further detail below. The mean difference between timepoints in left pupil size variability was −39.74 pixel/s (−52.51, −26.99) in the Cannabis Group and 0.58 pixel/s (−25.00, 26.16, *p* = 0.006) in the Control Group. The difference on the right side was −33.31 pixel/s (−47.32, −19.30) in the Cannabis Group and 6.44 pixel/s (−16.55, 29.42, *p* = 0.002) in the Control Group.

**Figure 3:**
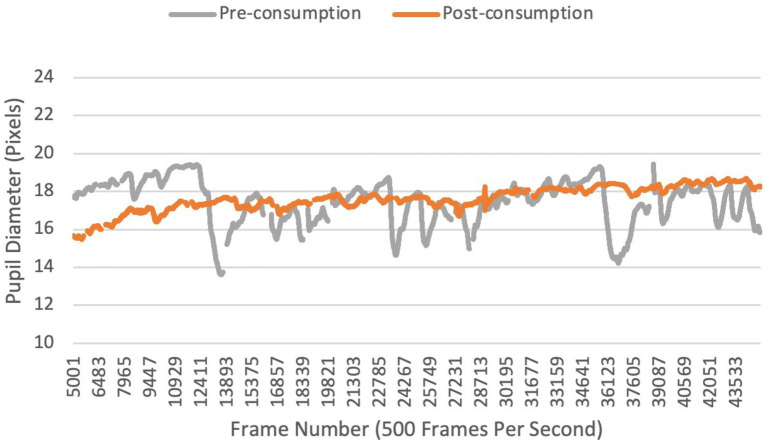
Continuous recording of pupil size before and after inhaling cannabis in a single participant.

Figure 3 presents the mean pupil size of a single participant during the same part of the test before and after consuming cannabis. This figure aims to visually represent a reduction in pupil size variability. On visual inspection of the continuous pupil size recording, we can see that pupil size is increasing and decreasing when they are watching a movie clip before consumption but is relatively flat after consumption. No differences were seen in pupil size variability in either the right (*p* = 0.231) or left (*p* = 0.647) pupil between timepoints in the Control Group, suggesting that watching the video for the second time did not affect pupil size variability.

The correlation between pupil size variability and demographic and cannabis-related factors when participants were not (Cannabis Pre-consumption + Controls) and were (Cannabis Post-consumption) high are presented in Table 3. Pupil size variability did not correlate with any demographic variable at baseline. After consumption, reduced variability in the left pupil was associated with higher cannabis use history and was showing a trend towards significance in the right pupil. Reduced left pupil size variability also weakly correlated with increasing THC concentration and was approaching statistical significance for right pupil size variability (*p* = 0.057). Pupil size variability did not show any trend towards correlation with self-reported level of euphoria (*p* = 0.917, 0.822).

**Table 3:** Correlation of pupil size variability with demographic and cannabis-related variables.

	Not High (*n* = 189)		High (*n* = 159)	
	Right pupil	Left pupil	Right pupil	Left pupil
Age	0.032 (0.672)	0.043 (0.569)	0.102 (0.213)	0.114 (0.164)
Sex	0.061 (0.408)	0.088 (0.229)	0.091 (0.254)	0.023 (0.771)
Race	0.040 (0.588)	0.138 (0.060)	−0.052 (0.516)	0.027 (0.733)
Hispanic	−0.104 (0.156)	−0.027 (0.715)	−0.120 (0.131)	−0.063 (0.428)
Cannabis use frequency	−0.134 (0.068)	−0.093 (0.205)	−0.152 (0.056)	**−0.210 (0.008)**
Self-reported Euphoria	–	–	0.008 (0.917)	−0.018 (0.822)
THC percent	–	–	−0.156 (0.057)	**−0.202 (0.013)**
Type of Cannabis	–	–	0.036 (0.654)	0.039 (0.633)
Tobacco	–	–	−0.009 (0.910)	−0.015 (0.850)

Bolded values indicate a significant finding; data is presented as Spearman’s correlation coefficient (*p*-values).

## Discussion

Our exploratory study has informative results which contributes to the literature on the effects of recent cannabis inhalation on eye function (Stark et al., 2003; Newmeyer et al., 2017; Campobasso et al., 2020; Shahidi Zandi et al., 2021). Participants in our study consisted of a heavy cannabis user population, with over 60% reporting they were daily users and used product with high THC concentration, and had equal distribution of males and females. At baseline, when the Cannabis Group had not consumed any cannabis (at least 6 h for inhaled and 24 h for edibles), no statistically significant differences were seen in any physiological or neurobehavioral assessments, which provides some internal validation that these participants were, on average, sober. This internal validation was crucial since we were not able to perform any biofluid assessments due to limitations in funding and could not confirm that users had abstained. After cannabis consumption, participants had significantly higher pulses and blood pressures, performed worse on the PVSAT, and a higher proportion had abnormal clues on the balance tests. These physiological and behavioral changes were expected since cannabis causes sympathetic activation (Borgelt et al., 2013) and Drug Recognition Experts use increased pulse and blood pressures as part of their cannabis-induced impairment examination (Hartman et al., 2016). Similarly, the DAT was developed to be sensitive for substance-related impairment (Burns, 2003) and we also found that participants had more abnormal clues after consuming cannabis.

Regarding differences in eye function, we were able to identify several parameters related to pupil dynamics and mismatched gaze, that were significantly different after recent cannabis consumption and were not different when compared to controls at baseline. For variables relating to gaze, although several differences were observed between Cannabis Post-consumption and Controls, these variables were already close to being different before consumption which suggests that cannabis use has a prolonged duration of effect on these parameters. Regarding pupil size, we found that mean pupil size increased after smoking cannabis, which is similar to what others have found (Hepler et al., 1972; Brown et al., 1977). However, pupil sizes were already larger than the Control Group before consumption which supports prior research supporting the prolonged effects of cannabis on pupil size (Huestegge et al., 2009, 2010). Research reports that pupil size has a strong (Pearsons r = 0.81–0.86) correlation with blood THC level (Shahidi Zandi et al., 2021). The rate at which THC is excreted from the body depends on several demographic and cannabis-related variables (Chayasirisobhon, 2020; Sideris et al., 2024), and THC metabolites persist in blood longer than duration of euphoria (Spindle et al., 2021). Although it had been several hours since the Cannabis Group had consumed any cannabis, more than half of the sample self-reported to be daily users meaning that it was highly likely that there were still elevated levels of THC in their blood. We did not quantify THC in our study and are unable to perform the same comparison, however, a future exploratory analysis of the current dataset is planned that compares non-ocular assessments to ocular assessments.

The current study also found that cannabis inhalation immediately reduced pupil dilation and constriction speeds, which has been reported previously in the literature (Campobasso et al., 2020). However, our study has a novel finding that has not been described before. One of the most discriminant variables between groups after consumption was pupil size variability, which is the variance in the time domain as opposed to the variance in pupil size area (Shaffer and Ginsberg, 2017). Recent cannabis consumption caused a reduction in pupil size variability in our sample, and pupil size variability is directly under Autonomic Nervous System control (Kanakis and Rosen, 1977; Karemaker, 2017). When the eyes are watching a video or reading and paying attention, light enters the retina and is processed in several visual and cognitive centers in the brain, which sends feedback to adjust the size of the pupil in response to what is being seen (Partala and Surakka, 2003). Published research suggests that reduction in pupil size variability is directly proportional to working memory capacity (Aminihajibashi et al., 2019) and is also associated with fluid intelligence and attention control (Lu et al., 2021). The Cannabis Group also performed worse on the PVSAT after consumption which suggests their working memory was reduced. Reduction in pupil size variability could, therefore, be a consequence of cannabis-induced cognitive impairment and autonomic dysfunction and has the possibility of becoming an indirect physiological biomarker for recent consumption. Pupil size variability was not different in controls on repeat assessment which suggests it is not confounded by the learning effect. However, additional longitudinal studies need to be performed to see when this marker returns to normative values as well as identify if there are any parameters than can confound this, such as level of sleep deprivation (Shekari Soleimanloo et al., 2019) and alcohol consumption (Hartman et al., 2015). Based on data from our pilot study, we would require 48 participants in each group to validate our pupil size variability finding when comparing to cannabis users and 22 participants in each group when comparing to non-or infrequent cannabis users at 80% power and an alpha of 0.05 using a two-sided independent samples *t*-test.

There are several limitations to this study. The first limitation is that this study is at risk of bias. The primary author (MNH) is a paid advisor for the eye-tracking device that is used in this study and the eye-tracking company financially supported the study. Additionally, due to limitations in funding, we could not confirm using a blood or urine test that cannabis users were not under the influence of cannabis when they came to perform the baseline assessment. All participants said they had not consumed for several hours, but no member of the research team are trained Drug Recognition Experts so we are not qualified to perform this screening and had to take the participant’s word for it. Although we checked every container and recorded THC percentage, we were not able to calculate the volume THC consumed since we were not able to record mass consumed in vapor pens. A future exploratory analysis that only includes participants who consumed cannabis flower is planned to see if there are any eye tracking metrics that correlate with volume (percent X mass) of THC flower smoked. There is also no blinding in any part of our assessments, which can affect grading of subjective tests such as the balance tests. Another limitation is that we only used inhaled cannabis in our study. Edible cannabis use is common and should also be studied since the onset and duration of euphoria is much longer (Zipursky et al., 2020). We also cannot comment on how these parameters are affected with the co-use of alcohol, which is common (Ramaekers et al., 2009; Asbridge et al., 2012; Hartman et al., 2015). Lastly, although all participants were observed consuming cannabis and reported they were high, participants reported a range of self-reported euphoria (slightly high to very high). Cannabis affects people differently based on amount and potency consumed, tolerance, body size, smoking pattern (i.e., how much they inhale and over how long) (Macdonald et al., 2010; Spindle et al., 2021). A future exploratory analysis is planned that compares physiological and neurobehavioral assessments between levels of self-reported euphoria to see if there is anything that can explain this variance.

### Implications/recommendations for integration

This study only assesses the effect of recent inhaled cannabis consumption, and not impairment. There is no single gold-standard method to classify impairment, and these definitions are dependent on the context for assessment. Additionally, there are several causes for false positives on field sobriety testing which we did not account for, such as the presence of an intellectual disability or pathological causes of balance dysfunction. Future studies should attempt to control for them. Drug Recognition Experts are trained individuals who classify impairment using a multitude of behavioral, coordination, physiological and circumstantial assessments at multiple timepoints to make this classification (Hartman et al., 2016). Research that is focused on screening for cannabis-induced impairment (as opposed to recent use alone) should utilize trained experts to classify impairment and well as account for what can be integrated into current evaluation programs that are typically being conducted in an uncontrolled environment, i.e., on the roadside or within a police station.

## Conclusion

This pilot study has several findings that are relevant to developing cannabis screening tests. Even in heavy users, cannabis inhalation immediately caused greater mismatch in gaze and a reduction in pupil dynamics. These parameters were not different from cannabis naïve or infrequent users when cannabis users had abstained for several hours, suggesting these findings are not confounded by cannabis consumption history. The most discriminant parameter was a reduction in pupil size variability, which also negatively correlated with THC concentration of product used, and prior research has found reduced pupil size variability to be associated with reduced cognitive ability and working memory. This variable did not change in the control group between assessments suggesting it is a reliable finding, and was not associated with age, sex or race of the participant before or after consumption. Future studies should assess pupil size variability after cannabis consumption at multiple timepoints to see when this variable returns to normative values and see if it correlates with biofluid quantification of THC metabolites.

## Data availability statement

The raw data supporting the conclusions of this article will be made available by the authors, without undue reservation.

## Ethics statement

The studies involving humans were approved by WIRB-Copernicus Group, Inc. (also called WCG). The studies were conducted in accordance with the local legislation and institutional requirements. The participants provided their written informed consent to participate in this study.

## Author contributions

MoH: Conceptualization, Data curation, Formal analysis, Funding acquisition, Methodology, Project administration, Supervision, Writing – original draft, Writing – review & editing. DR: Data curation, Methodology, Writing – original draft, Writing – review & editing. MaH: Data curation, Investigation, Methodology, Writing – original draft, Writing – review & editing. FA: Formal analysis, Writing – original draft, Writing – review & editing. AI: Conceptualization, Data curation, Methodology, Writing – original draft, Writing – review & editing.
