# Supplementary material for: Effects of recent cannabis consumption on eye-tracking and pupillometry
Source: Front Neurosci. 2024 Apr 9;18:1358491. doi: 10.3389/fnins.2024.1358491 (PMC11036868; doi:10.3389/fnins.2024.1358491)
Supplement: Supplementary file 2 [file Data_Sheet_2.pdf]

Supplement File 2 for Effects of Recent Cannabis Consumption on Eye-tracking and Pupillometry

Figure e1. Right and left gaze during test

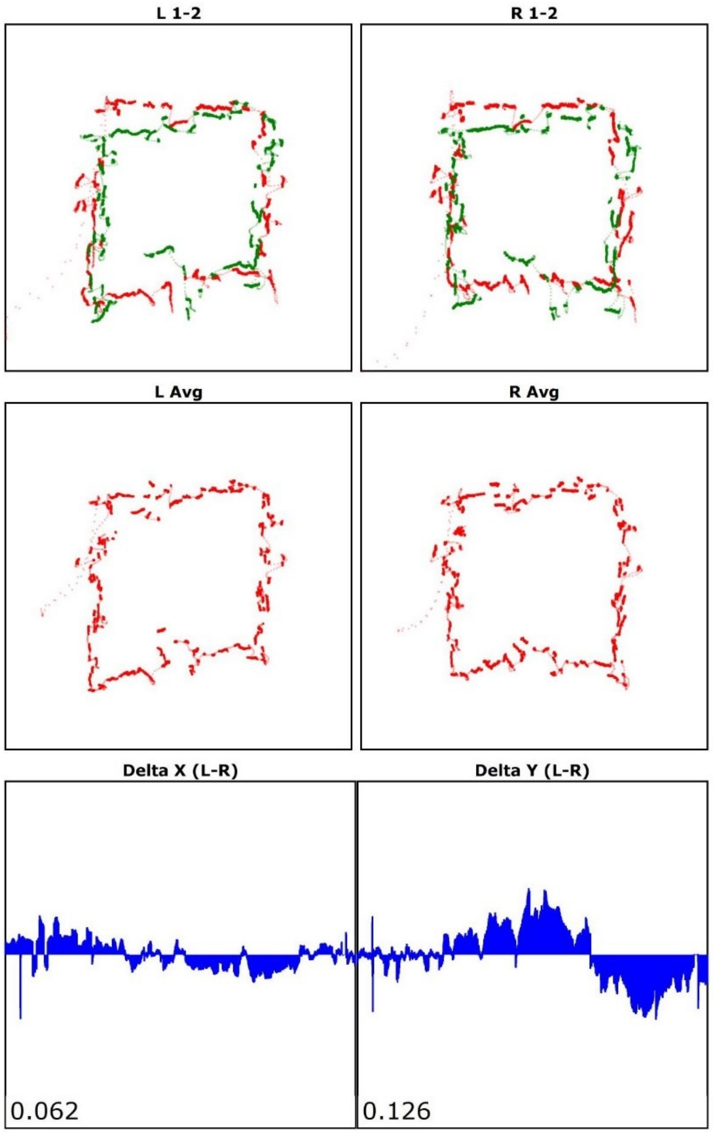

Sample summary report of eye tracking while watching a video moving in a square frame. The top figures are from the 1st and 2nd cycle, the middle figure is the average over two cycles, and the bottom figures are the difference between right and left pupils during the test. Calculated values included mean and variance of pupil position during each part of the frame (top, bottom, left and right).

Table e1. Comparison of all EyeBOX measurements between Control Baseline and Repeat assessment

	Control Baseline			Control Repeat			Cohen's d	p-value
	Mean	Lower CI	Upper CI	Mean	Lower CI	Upper CI		
Average Radial Distance Left Eye	0.0029	0.0026	0.0031	0.0028	0.0025	0.0031	0.075	0.824
Average Radial Distance Right Eye	0.0028	0.0025	0.003	0.0028	0.0025	0.003	0.012	0.871
Horizontal Saccade Travel Mean Left Eye	0.2181	0.192	0.2443	0.2536	0.2233	0.2839	-0.468	0.132
Horizontal Saccade Travel Mean Right Eye	0.2276	0.203	0.2522	0.2481	0.2148	0.2814	-0.261	0.433
Horizontal Saccade Travel Variance Left Eye	0.0174	0.0145	0.0204	0.0219	0.0144	0.0295	-0.295	0.679
Horizontal Saccade Travel Variance Right Eye	0.0184	0.0155	0.0213	0.0226	0.0142	0.0309	-0.262	0.889
Mean Path Departure Left Eye	0.092	0.0683	0.1157	0.0946	0.0723	0.1169	-0.042	0.976
Mean Path Departure Right Eye	0.0902	0.0723	0.108	0.1021	0.0767	0.1276	-0.203	0.636
Mean Saccade Travel Difference X axis	0.031	0.0269	0.0351	0.0287	0.0241	0.0333	0.195	0.451
Mean Saccade Travel Difference Y axis	0.044	0.037	0.0517	0.047	0.037	0.0570	-0.112	0.848
Mean Time to Peak Saccade Velocity Left Eye	15.53	14.54	16.52	15.56	13.88	17.24	-0.009	0.183
Mean Time to Peak Saccade Velocity Right Eye	14.72	14.23	15.22	18.11	13.98	22.24	-0.431	0.139
Mean Total X axis Movement Left Eye	2.43	2.42	2.44	2.411	2.395	2.40	0.477	0.183
Mean Total X axis Movement Right Eye	2.42	2.41	2.43	2.40	2.38	2.40	0.489	0.095
Mean Visual Area Left Eye	5.87	5.71	6.02	5.83	5.76	5.89	0.125	0.249
Mean Visual Area Right Eye	5.9	5.75	6.04	5.80	5.74	5.86	0.294	0.615
Mean Y axis Movement Left Eye	2.42	2.36	2.47	2.41	2.39	2.43	-0.014	0.101
Mean Y axis Movement Right Eye	2.44	2.38	2.49	2.41	2.39	2.43	0.189	0.859
Mean Y:X axis Aspect Ratio Left Eye	1.00	0.97	1.01	1.00	0.99	1.01	-0.183	**0.032**
Mean Y:X axis Aspect Ratio Right Eye	1.01	0.99	1.03	1.00	0.99	1.01	0.043	0.359
Orphan Saccade Ratio	0.1753	0.1368	0.2138	0.1574	0.1144	0.2005	0.164	0.391
Radial Distance from Bottom Frame Left Eye	0.0029	0.0026	0.0033	0.0028	0.0026	0.0031	0.130	0.690
Radial Distance from Bottom Frame Right Eye	0.003	0.0027	0.0033	0.0029	0.0026	0.0032	0.053	0.790
Radial Distance from Left Frame Left Eye	0.0027	0.0024	0.003	0.0027	0.0024	0.003	-0.103	0.690
Radial Distance from Left Frame Right Eye	0.0026	0.0023	0.0029	0.0027	0.0024	0.003	-0.116	0.615
Radial Distance from Right Frame Left Eye	0.0029	0.0026	0.0033	0.0028	0.0025	0.003	0.210	0.515
Radial Distance from Right Frame Right Eye	0.0028	0.0025	0.0031	0.0028	0.0025	0.0031	0.030	0.848
Radial Distance from Top Frame Left Eye	0.0029	0.0025	0.0033	0.0029	0.0024	0.0034	0.002	0.836
Radial Distance from Top Frame Right Eye	0.0027	0.0024	0.003	0.0027	0.0024	0.0029	0.059	0.802
Saccade Frequency Left Eye	12.53	9.69	15.36	12.80	9.68	15.92	-0.035	0.941
Saccade Frequency Right Eye	12.32	9.65	15	12.40	9.56	15.25	-0.011	0.790
Saccade Length Mean Left Eye	54.69	50.88	58.49	55.14	44.63	65.66	-0.022	0.114
Saccade Length Mean Right Eye	52.84	48.96	56.73	64.06	44.30	83.83	-0.294	0.941
Saccade Length Variance Left Eye	1430.43	646.71	2214.15	2416.14	-864.71	5697.00	-0.154	0.425
Saccade Length Variance Right Eye	862.54	530.56	1194.51	1984.79	-11.77	3981.35	-0.309	0.767
Saccade Peak Velocity Mean Left Eye	300.4	280.02	320.78	282.53	264.27	300.80	0.345	0.174
Saccade Peak Velocity Mean Right Eye	301.02	278.38	323.65	297.25	259.06	335.45	0.045	0.359
Variance Saccade Travel Difference X axis	0.0009	0.0005	0.0012	0.0008	0.0005	0.0011	0.109	0.926
Variance Saccade Travel Difference Y axis	0.0012	0.0008	0.0015	0.0026	0.0007	0.0045	-0.409	0.359
Vertical Saccade Travel Mean Left Eye	0.2304	0.209	0.2518	0.1891	0.1676	0.2107	0.717	**0.005***
Vertical Saccade Travel Mean Right Eye	0.2242	0.1979	0.2506	0.2041	0.1773	0.2308	0.284	0.198

Vertical Saccade Travel Variance Left Eye	0.024	0.0134	0.0345	0.0173	0.0096	0.0249	0.273	0.101
Vertical Saccade Travel Variance Right Eye	0.025	0.0126	0.0374	0.0209	0.0099	0.032	0.131	0.191
X axis Variance Left Frame Left Eye	0.0122	0.0008	0.0236	0.0105	0.0075	0.0136	0.076	0.214
X axis Variance Left Frame Right Eye	0.0066	0.005	0.0081	0.0101	0.0076	0.0126	-0.645	0.133
X axis Variance Right Frame Left Eye	0.0084	0.0061	0.0107	0.0079	0.0056	0.0102	0.078	0.595
X axis Variance Right Frame Right Eye	0.0092	0.0072	0.0113	0.0086	0.0062	0.0111	0.095	0.367
Y axis Variance Bottom Frame Left Eye	0.0188	0.0127	0.0248	0.0194	0.012	0.0268	-0.033	0.802
Y axis Variance Bottom Frame Right Eye	0.0182	0.0125	0.0238	0.0211	0.0147	0.0275	-0.180	0.391
Y axis Variance Top Frame Left Eye	0.0185	0.0079	0.0292	0.0132	0.0085	0.0178	0.244	0.261
Y axis Variance Top Frame Right Eye	0.0125	0.0097	0.0153	0.0132	0.0101	0.0164	-0.095	0.918
Left Pupil Constriction Speed Mean	0.0928	0.0836	0.1019	0.0943	0.0855	0.1031	-0.064	0.767
Left Pupil Constriction Speed Variability	117.35	108.59	126.11	120.67	109.55	131.78	-0.124	0.848
Left Pupil Dilation Speed Mean	0.0812	0.074	0.0885	0.0805	0.0747	0.0862	0.045	0.848
Left Pupil Dilation Speed Variability	100.32	92.11	108.53	102.16	92.93	111.39	-0.079	0.953
Left Pupil Size Variability	268.67	244.95	292.38	269.24	248.67	289.81	-0.010	0.647
Maximum Left Pupil Size	17.71	16.41	19.01	18.465	16.742	20.188	-0.185	0.574
Maximum Right Pupil Size	17.64	16.49	18.78	17.3416	16.291	18.3922	0.100	0.859
Mean Left Pupil Size	15.49	14.18	16.79	15.1888	14.0077	16.3698	0.090	0.871
Mean Right Pupil Size	15.29	14.14	16.45	14.9202	13.8444	15.9961	0.125	0.723
Minimum Left Pupil Size	12.98	11.74	14.21	12.5457	11.5592	13.5323	0.144	0.953
Minimum Right Pupil Size	12.7	11.61	13.79	11.921	10.8443	12.9978	0.268	0.433
Right Pupil Constriction Speed Mean	0.0945	0.0853	0.1037	0.0956	0.0883	0.1029	-0.051	0.544
Right Pupil Constriction Speed Variability	120.06	109.3	130.83	127.7386	114.7829	140.6943	-0.119	0.274
Right Pupil Constriction Speed Variance	0.0093	0.0072	0.0114	0.0127	0.0083	0.0171	-0.364	0.268
Right Pupil Dilation Speed Mean	0.0834	0.0767	0.0901	0.0827	0.0776	0.0878	0.044	0.871
Right Pupil Dilation Speed Variability	101.64	93.21	110.07	107.99	96.68	119.29	-0.238	0.506
Right Pupil Dilation Speed Variance	0.0049	0.0041	0.0057	0.0066	0.0046	0.0086	-0.398	0.564
Right Pupil Size Variability	270.18	246.67	293.68	276.61	260.26	292.96	-0.119	0.231
Mean Difference Pupil Size	0.9668	0.7644	1.1691	1.0121	0.8126	1.2116	-0.084	0.712
Mean Difference Pupil Speed	-0.0001	-0.0008	0.0006	-0.0005	-0.0014	0.0003	0.201	0.848

*Bolded values indicates a p-value < 0.05; * indicates a significant finding after correcting for multiple comparisons (p < 0.017).*

Table e2a. Groupwise gaze metrics from EyeBOX, mean values

	Cannabis Pre			Cannabis Post			Control Baseline		
	**Mean**	**Lower CI**	**Upper CI**	**Mean**	**Lower CI**	**Upper CI**	**Mean**	**Lower CI**	**Upper CI**
Average Radial Distance Left Eye	0.0031	0.0029	0.0033	0.0034	0.0031	0.0036	0.0029	0.0026	0.0031
Average Radial Distance Right Eye	0.0031	0.0028	0.0034	0.0033	0.0031	0.0035	0.0028	0.0025	0.003
Horizontal Saccade Travel Mean Left Eye	0.255	0.2395	0.2704	0.2428	0.23	0.2557	0.2181	0.192	0.2443
Horizontal Saccade Travel Mean Right Eye	0.2539	0.2395	0.2683	0.2488	0.237	0.2607	0.2276	0.203	0.2522
Horizontal Saccade Travel Variance Left Eye	0.0375	0.0265	0.0484	0.0233	0.02	0.0266	0.0174	0.0145	0.0204
Horizontal Saccade Travel Variance Right Eye	0.0368	0.0280	0.0456	0.023	0.0194	0.0266	0.0184	0.0155	0.0213
Mean Path Departure Left Eye	0.1582	0.1318	0.1845	0.1464	0.1233	0.1695	0.0920	0.0683	0.1157
Mean Path Departure Right Eye	0.1497	0.1238	0.1756	0.1338	0.1161	0.1515	0.0902	0.0723	0.1080
Mean Saccade Travel Difference X axis	0.0351	0.032	0.0381	0.0394	0.0357	0.043	0.0310	0.0269	0.0351

Mean Saccade Travel Difference Y axis	0.0520	0.0457	0.0582	0.0615	0.0532	0.0698	0.0444	0.037	0.0517
Time to Peak Saccade Velocity Left Eye	16.63	15.48	17.77	16.75	15.92	17.59	15.53	14.54	16.52
Time to Peak Saccade Velocity Right Eye	16.44	15.69	17.18	17.11	16.26	17.97	14.72	14.23	15.22
Mean Total X axis Movement Left Eye	2.39	2.36	2.42	2.43	2.40	2.46	2.43	2.42	2.44
Mean Total X axis Movement Right Eye	2.39	2.36	2.42	2.41	2.39	2.43	2.42	2.41	2.43
Mean Visual Area Left Eye	5.78	5.67	5.89	5.82	5.66	5.99	5.87	5.71	6.02
Mean Visual Area Right Eye	5.8	5.71	5.88	5.9	5.77	6.02	5.9	5.75	6.04
Mean Y axis Movement Left Eye	2.4	2.36	2.44	2.38	2.31	2.45	2.42	2.36	2.47
Mean Y axis Movement Right Eye	2.41	2.38	2.45	2.44	2.4	2.48	2.44	2.38	2.49
Mean Y:X axis Aspect Ratio Left Eye	1.02	0.97	1.06	0.97	0.94	1.01	1.00	0.97	1.01
Mean Y:X axis Aspect Ratio Right Eye	1.00	0.98	1.02	1.01	1.00	1.03	1.01	0.99	1.03
Orphan Saccade Ratio	0.2161	0.1897	0.2424	0.2293	0.204	0.2545	0.1753	0.1368	0.2138
Radial Distance from Bottom Frame Left Eye	0.0033	0.003	0.0036	0.0034	0.0031	0.0037	0.0029	0.0026	0.0033
Radial Distance from Bottom Frame Right Eye	0.0032	0.0029	0.0035	0.0036	0.0032	0.0039	0.003	0.0027	0.0033
Radial Distance from Left Frame Left Eye	0.003	0.0027	0.0032	0.0034	0.0031	0.0036	0.0027	0.0024	0.003
Radial Distance from Left Frame Right Eye	0.003	0.0027	0.0034	0.0033	0.003	0.0035	0.0026	0.0023	0.0029
Radial Distance from Right Frame Left Eye	0.0031	0.0028	0.0034	0.0033	0.0031	0.0035	0.0029	0.0026	0.0033
Radial Distance from Right Frame Right Eye	0.0034	0.0027	0.0034	0.0034	0.0032	0.0037	0.0028	0.0025	0.0031
Radial Distance from Top Frame Left Eye	0.0030	0.0028	0.0033	0.0033	0.0031	0.0035	0.0029	0.0025	0.0033
Radial Distance from Top Frame Right Eye	0.0032	0.0029	0.0036	0.0032	0.003	0.0034	0.0027	0.0024	0.003
Saccade Frequency Left Eye	12.55	11.08	14.03	12.54	11.07	14.02	12.53	9.69	15.36
Saccade Frequency Right Eye	13.19	11.67	14.71	12.61	11.05	14.17	12.32	9.65	15.00
Saccade Length Mean Left Eye	55.34	53.02	57.67	57.76	55.09	60.43	54.69	50.88	58.49
Saccade Length Mean Right Eye	53.97	51.89	56.06	59.72	56.67	62.77	52.84	48.96	56.73
Saccade Length Variance Left Eye	1339.81	941.64	1737.98	1580.9	1099.38	2062.42	1430.4	646.71	2214.15
Saccade Length Variance Right Eye	1365.28	990.93	1739.64	2227.2	1547.28	2907.09	862.54	530.56	1194.51
Saccade Peak Velocity Mean Left Eye	301.76	288.99	314.54	296.21	286.76	305.66	300.4	280.02	320.78
Saccade Peak Velocity Mean Right Eye	306.51	290.48	322.55	290.1	281.18	299.02	301.02	278.38	323.65
Variance Saccade Travel Difference X axis	0.0016	0.0011	0.0021	0.0025	0.0014	0.0035	0.0009	0.0005	0.0012
Variance Saccade Travel Difference Y axis	0.0044	0.0028	0.0061	0.005	0.0027	0.0073	0.0012	0.0008	0.0015
Vertical Saccade Travel Mean Left Eye	0.2254	0.2146	0.2362	0.2203	0.2049	0.2357	0.2304	0.209	0.2518
Vertical Saccade Travel Mean Right Eye	0.2308	0.2178	0.2438	0.2097	0.1972	0.2222	0.2242	0.1979	0.2506
Vertical Saccade Travel Variance Left Eye	0.0257	0.0211	0.0303	0.0292	0.023	0.0353	0.024	0.0134	0.0345
Vertical Saccade Travel Variance Right Eye	0.0332	0.0257	0.0407	0.0268	0.02	0.0335	0.025	0.0126	0.0374
X axis Variance Left Frame Left Eye	0.0199	0.0094	0.0303	0.0276	-0.0002	0.0555	0.0122	0.0008	0.0236
X axis Variance Left Frame Right Eye	0.0193	0.0082	0.0304	0.0131	0.0096	0.0167	0.0066	0.005	0.0081
X axis Variance Right Frame Left Eye	0.0233	0.009	0.0376	0.0158	0.0094	0.0222	0.0084	0.0061	0.0107
X axis Variance Right Frame Right Eye	0.014	0.0077	0.0203	0.0223	0.0096	0.0351	0.0092	0.0072	0.0113
Y axis Variance Bottom Frame Left Eye	0.0353	0.0268	0.0438	0.0403	0.0319	0.0487	0.0188	0.0127	0.0248
Y axis Variance Bottom Frame Right Eye	0.0299	0.0225	0.0373	0.039	0.0204	0.0576	0.0182	0.0125	0.0238
Y axis Variance Top Frame Left Eye	0.022	0.0118	0.0321	0.0291	0.0154	0.0428	0.0185	0.0079	0.0292
Y axis Variance Top Frame Right Eye	0.0256	0.0151	0.0361	0.0225	0.0126	0.0324	0.0125	0.0097	0.0153

Table e2b. Groupwise gaze metrics from EyeBOX, effect sizes and p-values

	Pre vs Control		Post vs Control		Pre vs Post	
	**Cohen's d**	**p-value**	**Cohen's d**	**p-value**	**Cohen's d**	**p-value**
Average Radial Distance Left Eye	0.169	0.635	0.402	**0.029**	-0.175	**0.006***
Average Radial Distance Right Eye	0.208	0.573	0.485	**0.022**	-0.125	**0.006***
Horizontal Saccade Travel Mean Left Eye	0.392	0.056	0.312	0.147	0.135	0.334
Horizontal Saccade Travel Mean Right Eye	0.299	0.124	0.286	0.155	0.061	0.609
Horizontal Saccade Travel Variance Left Eye	0.318	0.222	0.307	0.372	0.278	0.493
Horizontal Saccade Travel Variance Right Eye	0.363	0.238	0.221	0.928	0.327	0.043
Mean Path Departure Left Eye	0.427	**0.050**	0.405	**0.038**	0.076	0.899
Mean Path Departure Right Eye	0.393	0.103	0.426	0.056	0.113	0.833
Mean Saccade Travel Difference X axis	0.226	0.541	0.389	0.080	-0.202	0.103
Mean Saccade Travel Difference Y axis	0.206	0.648	0.355	0.055	-0.207	**0.013***
Time to Peak Saccade Velocity Left Eye	0.162	0.924	0.248	0.375	-0.020	0.132
Time to Peak Saccade Velocity Right Eye	0.394	0.054	0.477	**0.017***	-0.133	0.343
Mean Total X axis Movement Left Eye	-0.191	0.152	0.002	0.729	-0.194	**0.012***
Mean Total X axis Movement Right Eye	-0.148	0.453	-0.046	0.782	-0.132	0.145
Mean Visual Area Left Eye	-0.132	0.286	-0.039	0.544	-0.055	0.580
Mean Visual Area Right Eye	-0.184	0.519	0.000	0.729	-0.141	0.828
Mean Y axis Movement Left Eye	-0.054	0.108	-0.078	0.869	0.055	**0.028**
Mean Y axis Movement Right Eye	-0.133	0.420	0.007	0.982	-0.127	0.250
Mean Y:X axis Aspect Ratio Left Eye	0.086	0.105	-0.092	0.830	0.163	**0.006***
Mean Y:X axis Aspect Ratio Right Eye	-0.062	0.503	0.032	0.906	-0.092	0.288
Orphan Saccade Ratio	0.256	0.431	0.352	0.097	-0.080	0.227
Radial Distance from Bottom Frame Left Eye	0.177	0.757	0.282	0.149	-0.078	0.052
Radial Distance from Bottom Frame Right Eye	0.122	0.724	0.311	0.208	-0.200	**0.008***
Radial Distance from Left Frame Left Eye	0.216	0.412	0.476	**0.007***	-0.245	**0.002***
Radial Distance from Left Frame Right Eye	0.213	0.399	0.488	**0.011***	-0.119	**0.005***
Radial Distance from Right Frame Left Eye	0.087	0.956	0.295	0.144	-0.156	**0.009***
Radial Distance from Right Frame Right Eye	0.129	0.757	0.394	**0.035**	0.006	**0.004***
Radial Distance from Top Frame Left Eye	0.142	0.637	0.308	**0.044**	-0.120	**0.011***
Radial Distance from Top Frame Right Eye	0.235	0.466	0.429	**0.020**	-0.004	**0.035**
Saccade Frequency Left Eye	0.003	0.581	0.002	0.610	0.001	0.991
Saccade Frequency Right Eye	0.093	0.991	0.030	0.553	0.059	0.362
Saccade Length Mean Left Eye	0.046	0.632	0.193	0.682	-0.153	0.094
Saccade Length Mean Right Eye	0.088	0.971	0.376	0.070	-0.346	**0.004***
Saccade Length Variance Left Eye	-0.037	0.715	0.052	0.942	-0.088	0.446
Saccade Length Variance Right Eye	0.230	0.767	0.347	0.250	-0.250	0.109
Saccade Peak Velocity Mean Left Eye	0.018	0.721	-0.071	0.726	0.078	0.944
Saccade Peak Velocity Mean Right Eye	0.057	0.667	-0.190	0.489	0.199	0.738
Variance Saccade Travel Difference X axis	0.234	0.495	0.281	0.092	-0.175	0.096
Variance Saccade Travel Difference Y axis	0.341	0.095	0.294	**0.007***	-0.043	0.097
Vertical Saccade Travel Mean Left Eye	-0.075	0.442	-0.110	0.132	0.061	0.171
Vertical Saccade Travel Mean Right Eye	0.081	0.842	-0.186	0.362	0.261	0.055
Vertical Saccade Travel Variance Left Eye	0.059	0.587	0.140	0.578	-0.103	0.980

Vertical Saccade Travel Variance Right Eye	0.181	0.556	0.043	0.764	0.143	0.119
X axis Variance Left Frame Left Eye	0.123	0.088	0.095	0.088	-0.058	0.917
X axis Variance Left Frame Right Eye	0.197	0.064	0.315	**0.011***	0.118	0.299
X axis Variance Right Frame Left Eye	0.179	0.263	0.197	0.098	0.107	0.430
X axis Variance Right Frame Right Eye	0.130	0.834	0.175	0.135	-0.130	**0.007***
Y axis Variance Bottom Frame Left Eye	0.331	**0.038**	0.434	**0.009***	-0.093	0.271
Y axis Variance Bottom Frame Right Eye	0.270	0.069	0.191	**0.042**	-0.100	0.365
Y axis Variance Top Frame Left Eye	0.057	0.763	0.130	0.847	-0.093	0.370
Y axis Variance Top Frame Right Eye	0.215	0.448	0.173	0.541	0.048	0.701

*Bolded values indicates a p-value < 0.05; * indicates a significant finding after correcting for multiple comparisons (p < 0.017).*

Table e3a. Groupwise pupil dynamics metrics from EyeBOX, mean values

	Cannabis Pre			Cannabis Post			Control Baseline		
	**Mean**	**Lower CI**	**Upper CI**	**Mean**	**Lower CI**	**Upper CI**	**Mean**	**Lower CI**	**Upper CI**
Left Pupil Constriction Speed Mean	0.089	0.084	0.0941	0.0735	0.0692	0.0778	0.0928	0.0836	0.1019
Left Pupil Constriction Speed Variability	129.49	120.69	138.3	125.38	117.88	132.88	117.35	108.59	126.11
Left Pupil Dilation Speed Mean	0.0767	0.0733	0.0801	0.0653	0.0619	0.0687	0.0812	0.074	0.0885
Left Pupil Dilation Speed Variability	109.72	102.92	116.51	110.49	103.89	117.1	100.32	92.11	108.53
Left Pupil Size Variability	247.85	235.22	260.47	204.98	194.55	215.4	268.67	244.95	292.38
Maximum Left Pupil Size	20.07	19.18	20.97	19.52	18.85	20.2	17.71	16.41	19.01
Maximum Right Pupil Size	19.33	18.52	20.15	19.34	18.55	20.12	17.64	16.49	18.78
Mean Left Pupil Size	17.2	16.57	17.83	17.26	16.63	17.89	15.49	14.18	16.79
Mean Right Pupil Size	16.87	16.19	17.54	17.03	16.35	17.7	15.29	14.14	16.45
Minimum Left Pupil Size	14.51	13.92	15.1	14.56	13.9	15.22	12.98	11.74	14.21
Minimum Right Pupil Size	14.19	13.55	14.82	14.27	13.63	14.92	12.7	11.61	13.79
Right Pupil Constriction Speed Mean	0.0886	0.0838	0.0935	0.0737	0.0696	0.0777	0.0945	0.0853	0.1037
Right Pupil Constriction Speed Variability	129.99	121.48	138.51	124.96	117.76	132.16	120.06	109.3	130.83
Right Pupil Constriction Speed Variance	0.0115	0.0094	0.0135	0.0095	0.008	0.011	0.0093	0.0072	0.0114
Right Pupil Dilation Speed Mean	0.0764	0.0732	0.0796	0.0669	0.0635	0.0703	0.0834	0.0767	0.0901
Right Pupil Dilation Speed Variability	110.17	103.43	116.9	113.78	106.47	121.09	101.64	93.21	110.07
Right Pupil Dilation Speed Variance	0.0061	0.0052	0.007	0.0068	0.0054	0.0082	0.0049	0.0041	0.0057
Right Pupil Size Variability	246.31	233.58	259.05	209.91	198.58	221.24	270.18	246.67	293.68
Mean Difference Pupil Size	1.2359	1.0195	1.4522	1.2707	1.1145	1.4268	0.9668	0.7644	1.1691
Mean Difference Pupil Speed	-0.0004	-0.0011	0.0003	-0.0008	-0.0016	0.0000	-0.0001	-0.0008	0.0006

Table e3b. Groupwise pupil dynamics metrics from EyeBOX, effect sizes and p-values

	Pre vs Control		Post vs Control		Pre vs Post	
	**Cohen's d**	**p-value**	**Cohen's d**	**p-value**	**Cohen's d**	**p-value**
Left Pupil Constriction Speed Mean	-0.121	0.271	-0.717	**<0.001***	0.523	**<0.001***
Left Pupil Constriction Speed Variability	0.233	0.71	0.179	0.760	0.079	0.415
Left Pupil Dilation Speed Mean	-0.213	0.298	-0.747	**<0.001***	0.530	**<0.001***

Left Pupil Dilation Speed Variability	0.232	0.544	0.256	0.520	-0.018	0.894
Left Pupil Size Variability	-0.268	0.092	-0.964	**<0.001***	0.582	**<0.001***
Maximum Left Pupil Size	0.438	**0.018**	0.434	**0.022**	0.109	0.926
Maximum Right Pupil Size	0.346	0.063	0.356	0.068	-0.001	0.850
Mean Left Pupil Size	0.439	**0.018**	0.451	**0.013***	-0.016	0.680
Mean Right Pupil Size	0.382	**0.040**	0.415	**0.025**	-0.036	0.578
Minimum Left Pupil Size	0.416	**0.018**	0.386	**0.028**	-0.011	0.756
Minimum Right Pupil Size	0.384	**0.044**	0.399	**0.035**	-0.021	0.607
Right Pupil Constriction Speed Mean	-0.196	0.161	-0.810	**<0.001***	0.525	**<0.001***
Right Pupil Constriction Speed Variability	0.195	0.496	0.112	0.904	0.100	0.364
Right Pupil Dilation Speed Mean	-0.351	0.108	-0.784	**<0.001***	0.452	**<0.001***
Right Pupil Dilation Speed Variability	0.212	0.519	0.277	0.447	-0.081	0.783
Right Pupil Size Variability	-0.305	**0.044**	-0.850	**<0.001***	0.475	**<0.001***
Mean Difference Pupil Size	0.210	0.837	0.323	0.204	-0.029	0.072
Mean Difference Pupil Speed	-0.066	0.578	-0.149	0.449	0.092	**0.030**

*Bolded values indicates a p-value < 0.05; * indicates a significant finding after correcting for multiple comparisons (p < 0.017).*
